# Enlargement of the Breasts in a Male: Atrophy of the Genital Organs

**Published:** 1845-05

**Authors:** 


*Enlargement of the Breasts in a Male: atrophy of the Genital Organs.*—A man, aged about 55, originally a quarryman, at Rochester, but afterwards one of the British Legion in Spain, was presented to the Society, having a remarkable developmnt of the breasts. He stated that he had been very healthy, had three children, the eldest 25, the youngest would have been between 8 and 9 years of age;that in an engagement in Spain, he tried to jump over a trench, but, being laden with his knapsack, failed, and fell with his chest against the opposite margin. He received a blow on the lower part of the spine, and falling some depth, received a violent blow on the occiput, which rendered him insensible. He was under the care of Mr. R. Alcock for some time. Since that period the mammæ have gradually increased in size, his voice has diminished in power, his beard has diminished, the right testicle has diminished to a very small size, and the left is still diminishing, and he has had no sexual desire since. He is partially paralysed on the right side, and moves about very much bent. Skin fair and soft; beard very thin and soft; voice weak; mammæ pendulous; nipples rather prominent; the glands very large and distinct; penis small and shrivelled; right testic having the feeling of condensed membrane; left of a moderate size, but not firm, and tender on pressure; the pelvic region round, more like that of a woman, not presenting the angular appearance of the male. He denies having ever had venereal disease, or taken any quantity of mercury.

Cases were mentioned of enlargement of the breasts in the male related in the "Philosophical Transactions," vol. 41; and in Captain Franklin's "Journey to the Polar Regions;" and of emasculation consequent upon blows or wounds of the occiput, related by Larrey, in his "Clinique Chirurgicale; and by Hennen in his "Military Surgery." There was no appearance of abscess iu the mammæ.—*Prov. Med. and Surg. Jour.*

